# Supplementary material for: Targeting of AKT1 by miR-143-3p Suppresses Epithelial-to-Mesenchymal Transition in Prostate Cancer
Source: Cells. 2023 Sep 5;12(18):2207. doi: 10.3390/cells12182207 (PMC10526992; doi:10.3390/cells12182207)
Supplement: Supplementary file 1 [file cells-12-02207-s001.zip › cells-2573134-supplementary.pdf]

Supplementary Figures

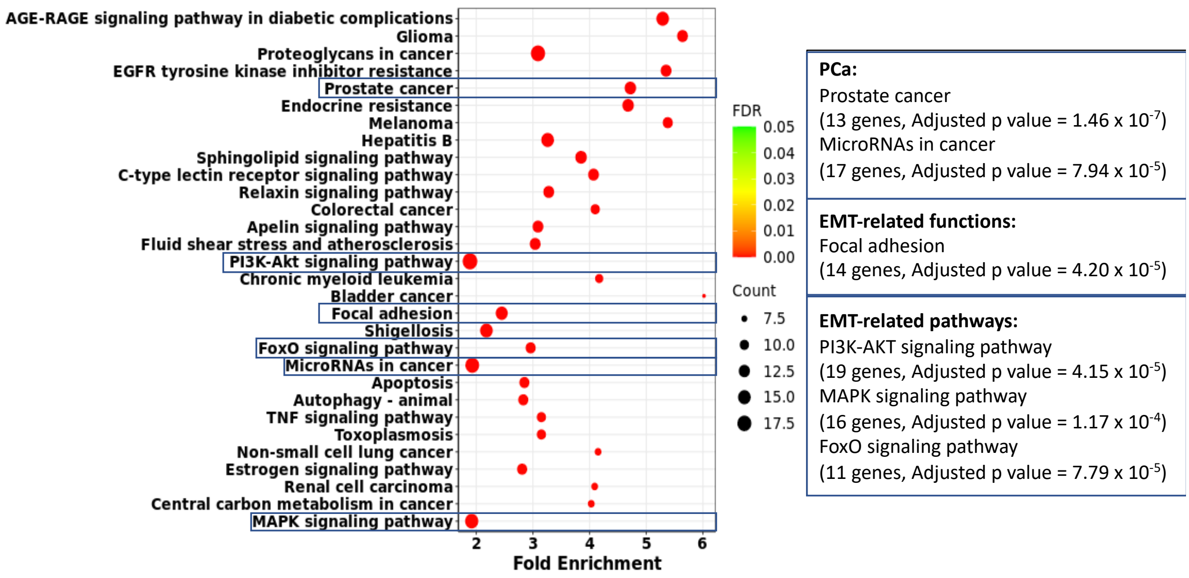

Figure S1. Bubble plot of KEGG functional enrichment analysis of miR-143-3p targets generated by CancerMIRNome.
EMT-related signaling pathways:
"PI3K-AKT": https://dbemt.bioinfo-minzhao.org/literature_highlight.cgi?gene=207 (accessed on 10 May 2023).
"MAPK": https://dbemt.bioinfo-minzhao.org/literature_highlight.cgi?gene=5594 (accessed on 10 May 2023).
"FoxO": https://dbemt.bioinfo-minzhao.org/literature_highlight.cgi?gene=2308. (accessed on 10 May 2023).
FDR = False Discovery Rate

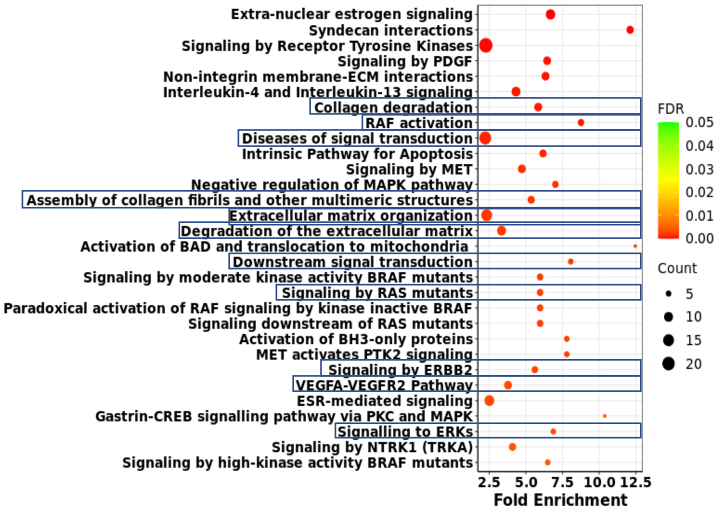

EMT-related functions:
Collagen degradation
(8 genes, Adjusted p value = 4.35×10^{-4})
Assembly of collagen fibrils and other multimeric structures
(7 genes, Adjusted p value = 1.72×10^{-3})
Extracellular matrix organization
(15 genes, Adjusted p value = 1.86×10^{-3})
Degradation of extracellular matrix
(10 genes, Adjusted p value = 1.97×10^{-5})

EMT-related pathways:
RAF activation
(32 genes, Adjusted p value = 5.85×10^{-4})
Diseases of signal transduction
(18 genes, Adjusted p value = 8.10×10^{-4})
Signaling by RAS mutants
(6 genes, Adjusted p value = 2.20×10^{-3})
Signaling by ERBB2
(6 genes, Adjusted p value = 2.75×10^{-5})
VEGFA-VEGFR2 Pathway
(8 genes, Adjusted p value = 3.07×10^{-3})
Signaling to ERKs
(5 genes, Adjusted p value = 3.54×10^{-3})

Figure S2. Bubble plot of REACTOME functional enrichment analysis of miR-143-3p targets generated by CancerMIRNome.
EMT-related signaling pathways:
"RAF": https://dbemt.bioinfo-minzhao.org/literature_highlight.cgi?gene=5894 (accessed on 12 May 2023).
"RAS": https://dbemt.bioinfo-minzhao.org/literature_highlight.cgi?gene=3845 (accessed on 12 May 2023).
"ERBB3": https://dbemt.bioinfo-minzhao.org/literature_highlight.cgi?gene=2064 (accessed on 12 May 2023).
"VEGFA-VEGFR2": https://dbemt.bioinfo-minzhao.org/literature_highlight.cgi?gene=7422 (accessed on 12 May 2023).
"PI3K-AKT": https://dbemt.bioinfo-minzhao.org/literature_highlight.cgi?gene=207 (accessed on 12 May 2023).
FDR = False Discovery Rate

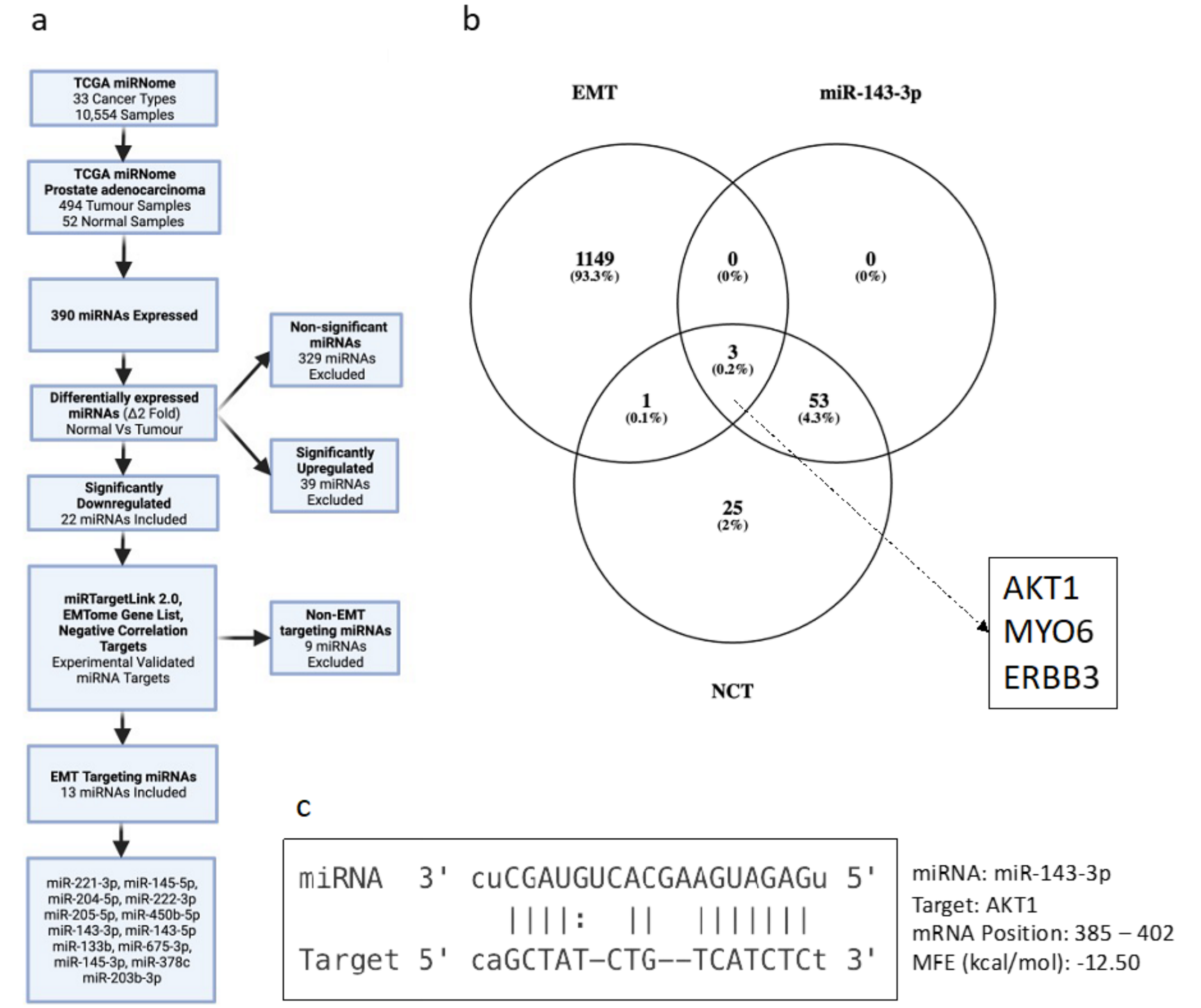

Figure S3. ***In silico*** **selection of downregulated miRNAs in prostate cancer associated with EMT.** (a) Selection process for identifying EMT associated miRNAs in prostate cancer. (b) Targets of the significantly downregulated miR-143-3p were predicted using miRTargetLink 2.0. Targets were cross-referenced to EMTome gene list and negative correlation targets in prostate cancer. (c) miR-TarBase computational prediction of miR-143-3p-AKT1 interaction. **EMT** = epithelial-to-mesenchymal transition; **NTC** = Negative Correlation Targets.

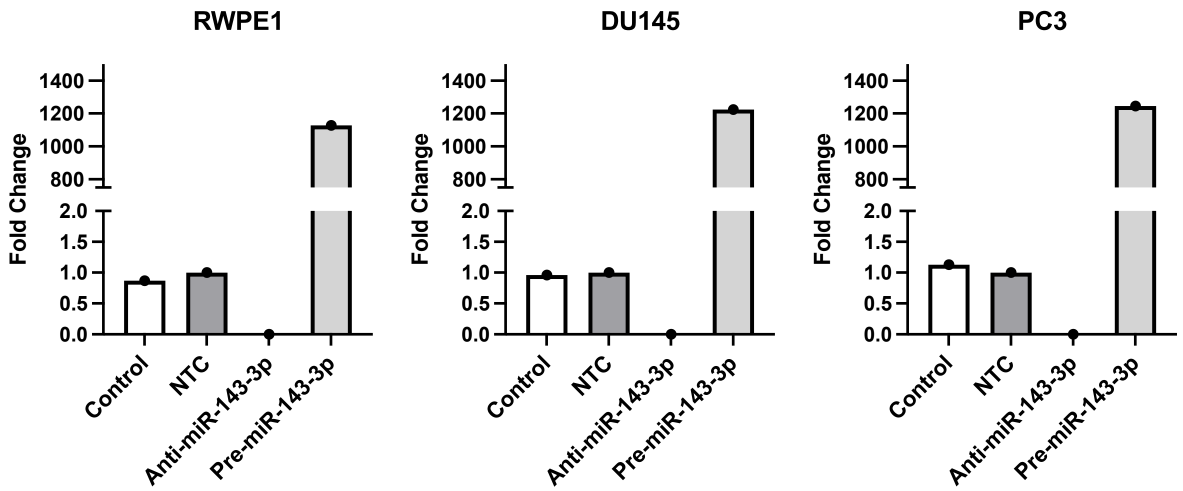

Figure S4: Confirmation of miR-143-3p overexpression and inhibition in prostate cell lines. (*n = 1*, housekeeping: snRNA U6).

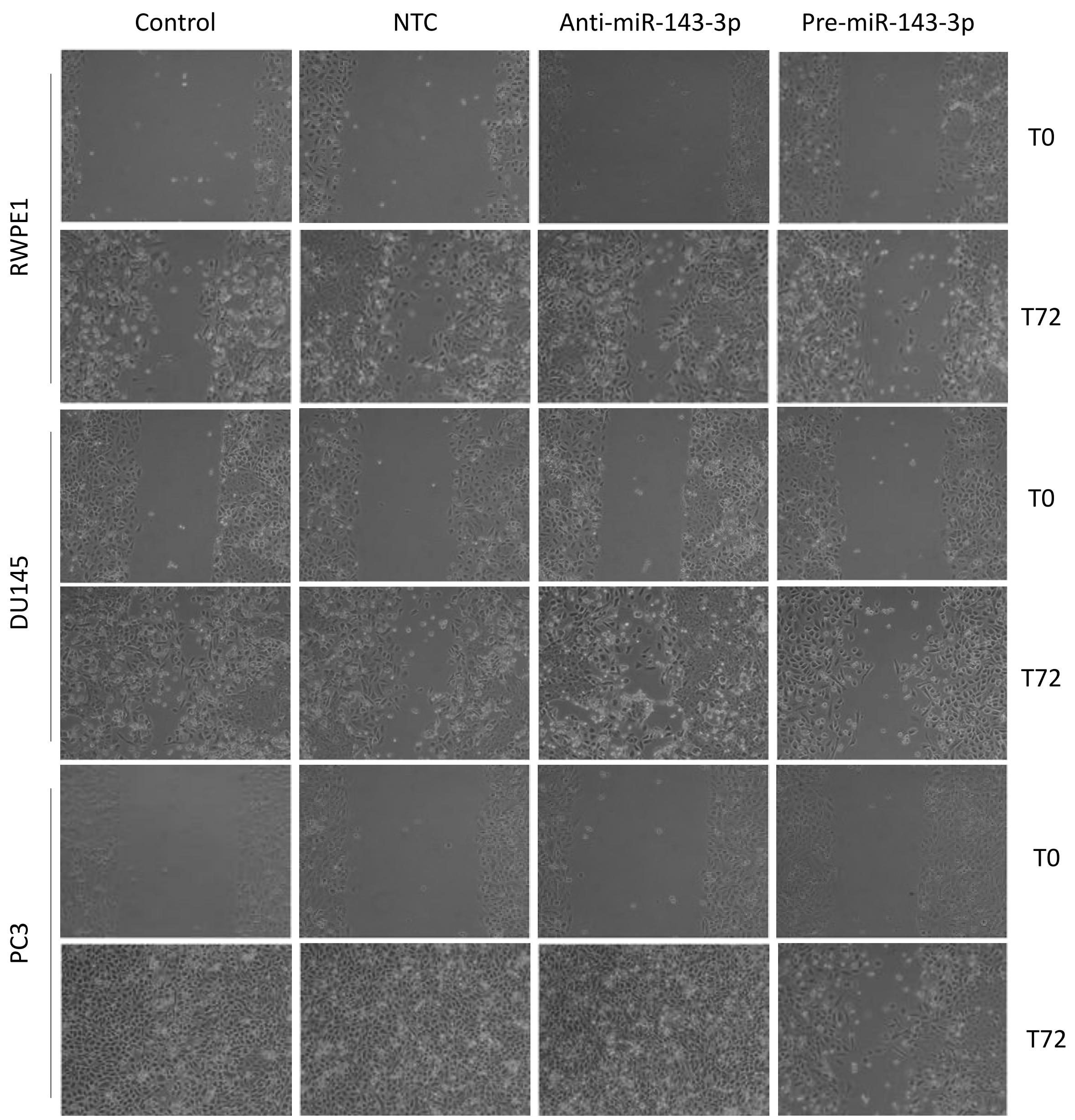

Figure S5: Representative images of wound-healing assay. miR-143-3p controls migratory capacity of RWPE1, DU145 and PC3 cell lines. Scratch assay performed at 72 hours post transfection using ImageJ software to quantify wound closure %. Pre-miR-Neg, Anti-miR-143-3p, Pre-miR-143-3p (25 nM). NTC = Non-targeting Control.

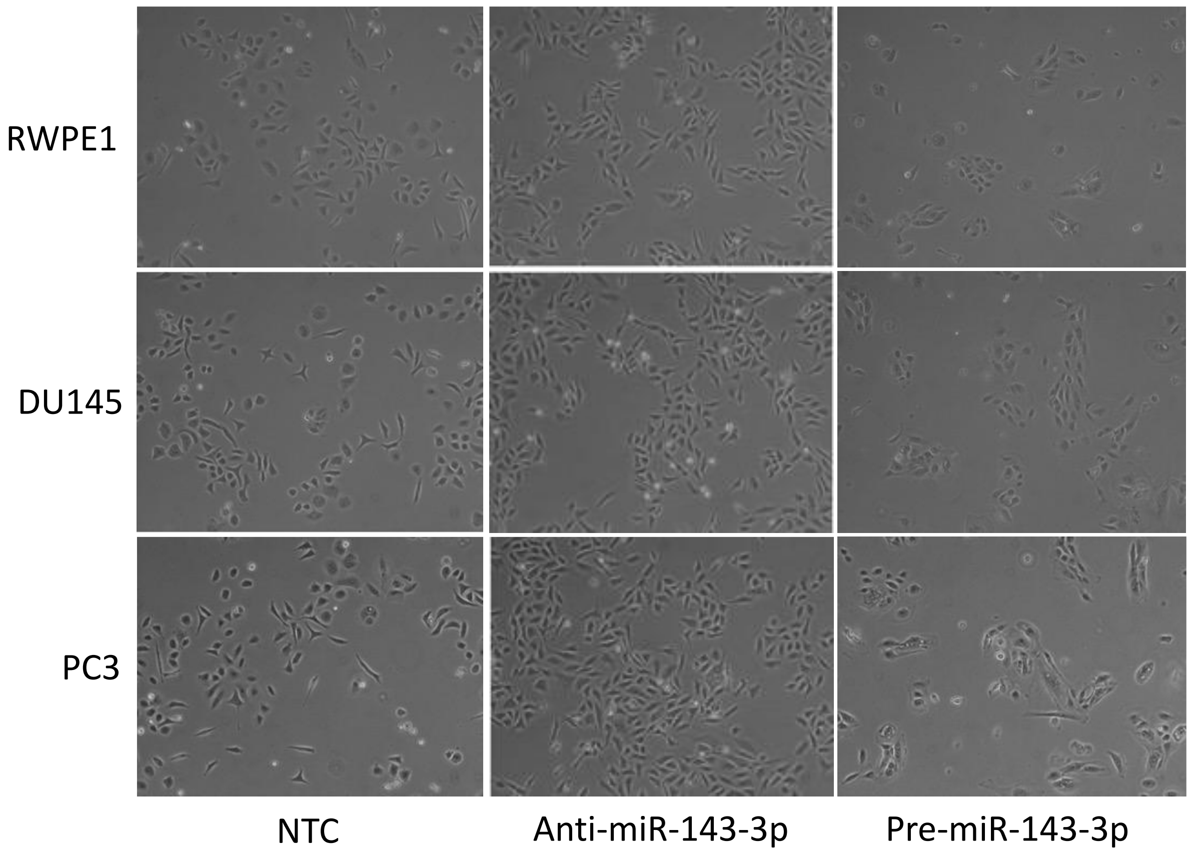

Figure S6: Representative images of clonogenicity assay. miR-143-3p alters colony forming capacity of RWPE1, DU145 and PC3 cell lines. Cell count performed using ImageJ software at 72 hours post transfection. Pre-miR-Neg, Anti-miR-143-3p, Pre-miR-143-3p (25 nM).

a

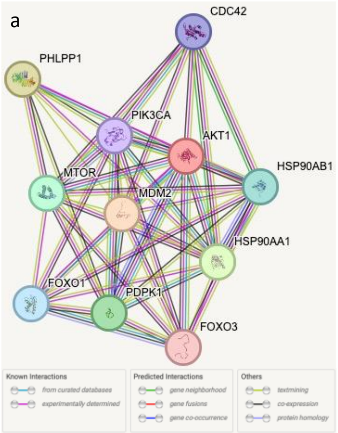

b

KEGG Pathways

pathway	description	count in network	strength	false discovery rate
hsa05215	Prostate cancer	8 of 97	2.17	2.70e-14
hsa04213	Longevity regulating pathway - multiple species	5 of 61	2.17	6.13e-09
hsa05213	Endometrial cancer	4 of 58	2.09	6.05e-07
hsa05223	Non-small cell lung cancer	4 of 68	2.02	1.05e-06
hsa04211	Longevity regulating pathway	5 of 87	2.01	2.74e-08
hsa01524	Platinum drug resistance	4 of 70	2.01	1.11e-06
hsa05214	Glioma	4 of 71	2.0	1.11e-06
hsa05212	Pancreatic cancer	4 of 71	2.0	1.11e-06
hsa04960	Aldosterone-regulated sodium reabsorption	2 of 37	1.99	0.0010
hsa04370	VEGF signaling pathway	3 of 56	1.98	4.08e-05
hsa01521	EGFR tyrosine kinase inhibitor resistance	4 of 77	1.97	1.30e-06
hsa04919	Thyroid hormone signaling pathway	6 of 120	1.95	2.29e-09
hsa04152	AMPK signaling pathway	6 of 120	1.95	2.29e-09
hsa04068	FoxO signaling pathway	6 of 126	1.93	2.29e-09
hsa04931	Insulin resistance	5 of 106	1.93	6.51e-08

Figure S7. Network analysis of AKT1 protein interactions. (**a**) Visualization of network by String-DB Version 12. (**b**) KEGG Pathway analysis of the AKT1 network is significantly associated with prostate cancer and pathways related to EMT.

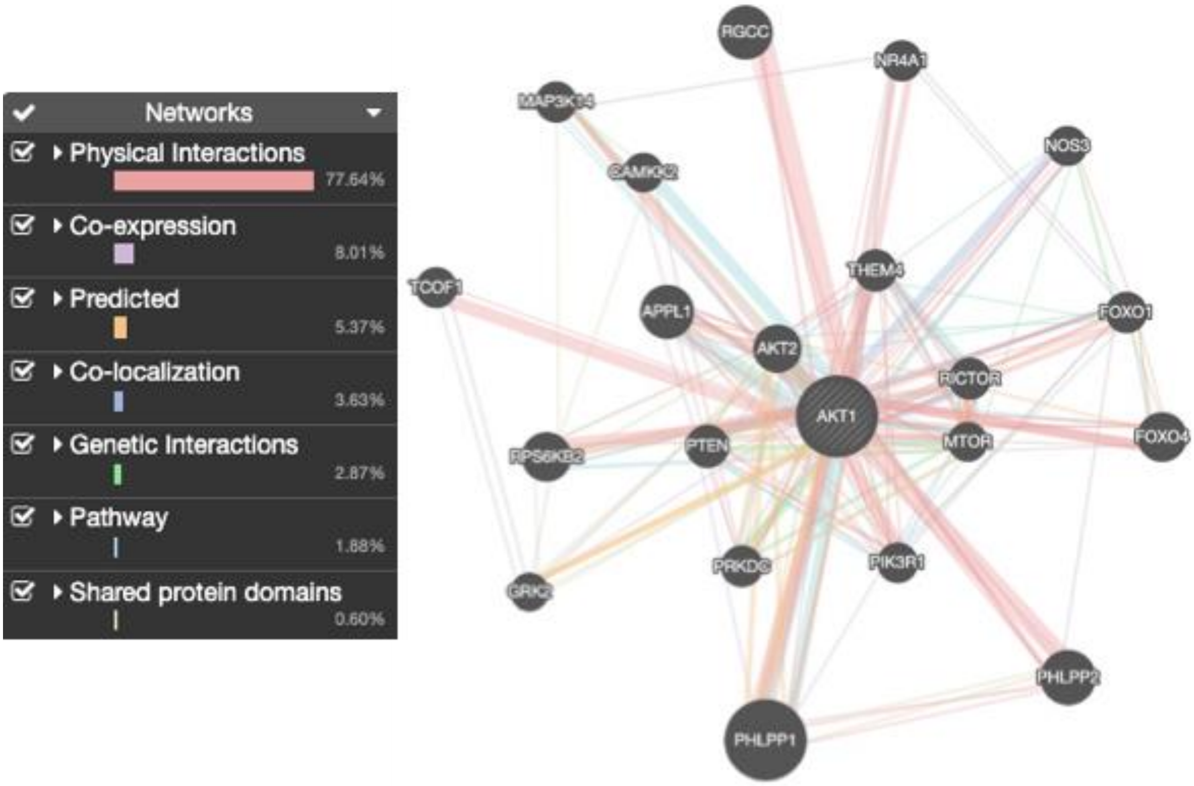

Figure S8. GeneMANIA network analysis of AKT1 gene interactions.

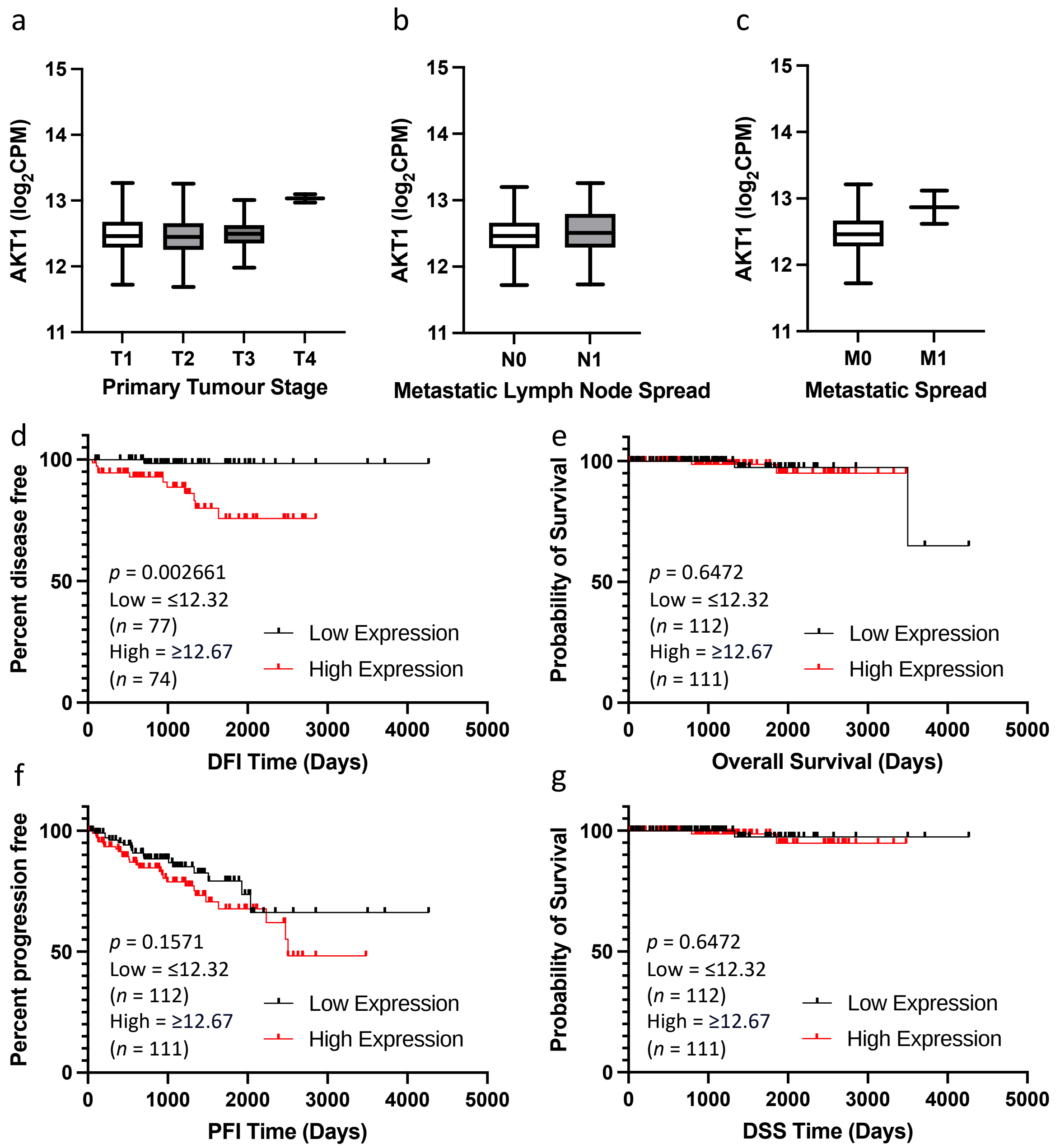

Figure S9. Correlation between AKT1 expression and clinicopathological markers of PCa progression. UCSC Xena analysis of TCGA PRAD samples shows AKT1 expression in (**a**) primary tumour stage, T1 (*n* = 152), T2 (*n* = 164), T3 (*n* = 50) and T4 (*n* = 2), (**b**) metastatic lymph node spread, N0 (*n* = 302) and N1 (*n* = 65) and (**c**) metastatic spread M0 (*n* = 365) and M1 (*n* = 2). For KM plots, patients were divided into quartiles based on AKT1 expression. (**d**) High expression of AKT1 was associated with significantly reduced DFI, Low = ≤12.32 (*n* = 77), High = ≥12.67 (*n* = 74). (**e**) No significant difference was observed between AKT1 expression and overall survival, Low = ≤12.32 (*n* = 112), High = ≥12.67 (*n* = 111) (**f**) No significant difference was observed between AKT1 expression and DSS, Low = ≤12.32 (*n* = 112), High = ≥12.67 (*n* = 111). (**g**) No significant difference was observed between AKT1 expression and PFI, Low = ≤12.32 (*n* = 112), High = ≥12.67 (*n* = 111). *p*-values for KM plots generated via log-rank (Mantel–Cox) tests. CPM = Copies per million; *n* = number; DFI = disease free interval; DSS = disease specific survival; PFI = Progression free interval.

Project	Tumor	Normal	AUC (95% CI)
TCGA−THYM	124	2	1 (0.98−1)
TCGA−READ	161	3	1 (0.99−1)
TCGA−CESC	307	3	1 (0.99−1)
TCGA−UCEC	538	33	0.98 (0.97−1)
TCGA−COAD	444	8	0.98 (0.95−1)
TCGA−LUSC	478	45	0.96 (0.94−0.98)
TCGA−BLCA	409	19	0.93 (0.88−0.98)
TCGA−PRAD	494	52	0.89 (0.85−0.93)
TCGA−KIRP	291	34	0.89 (0.86−0.93)
TCGA−KICH	66	25	0.89 (0.83−0.96)
TCGA−LUAD	513	46	0.85 (0.74−0.95)
TCGA−CHOL	36	9	0.85 (0.74−0.96)
TCGA−BRCA	1078	104	0.85 (0.82−0.88)
TCGA−HNSC	523	44	0.81 (0.74−0.88)
TCGA−STAD	436	41	0.8 (0.73−0.88)
TCGA−SKCM	97	2	0.73 (0.24−1)
TCGA−THCA	506	59	0.65 (0.58−0.71)
TCGA−PAAD	178	4	0.65 (0.26−1)
TCGA−LIHC	372	50	0.64 (0.58−0.71)
TCGA−ESCA	184	13	0.61 (0.42−0.8)
TCGA−KIRC	516	71	0.59 (0.53−0.65)
TCGA−PCPG	179	3	0.54 (0.2−0.87)

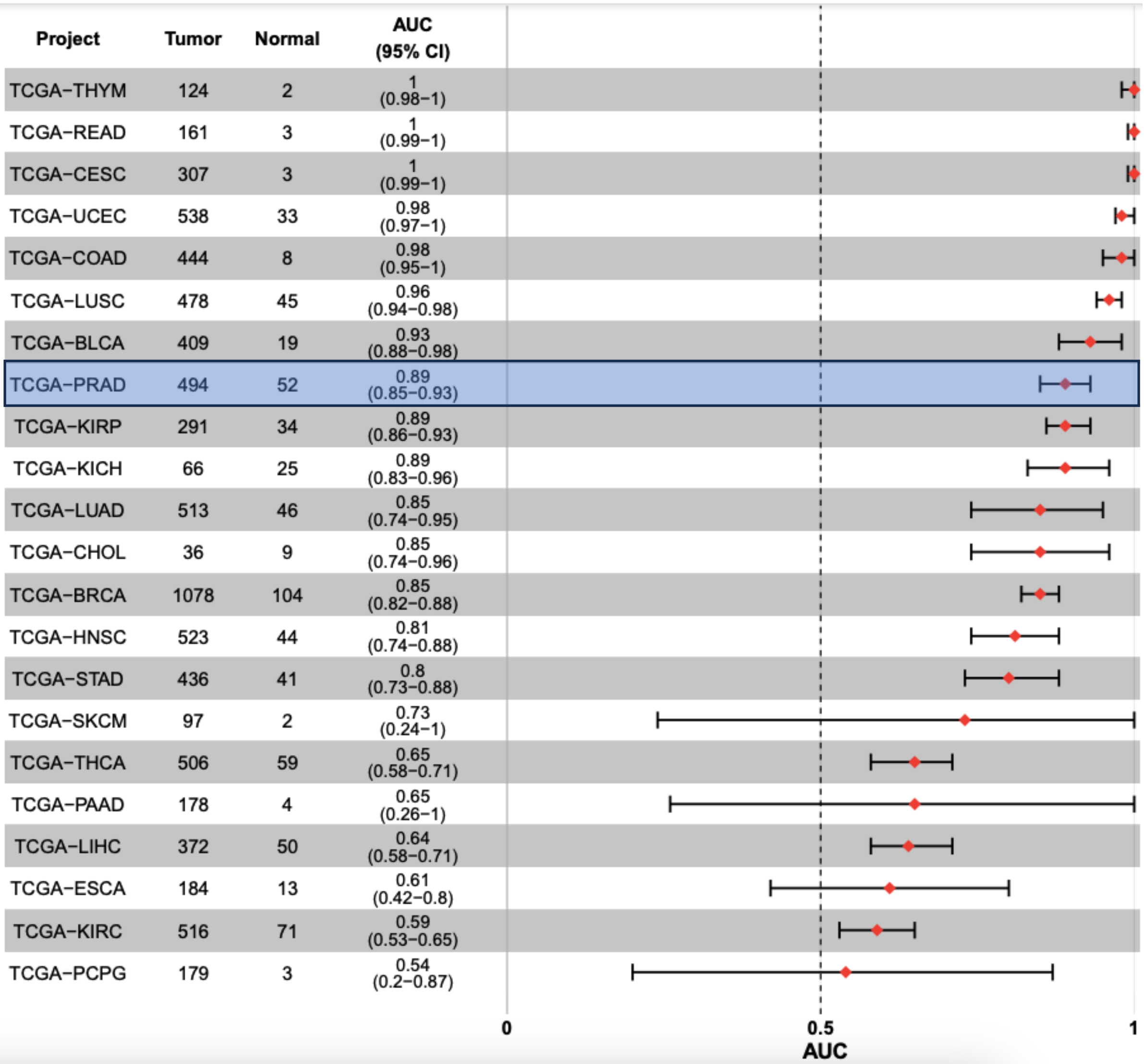

Figure S10. CancerMIRNome TCGA Pan- Cancer ranked forest plots of miR-143-3p ROC analysis shows miR-143-3p has significant diagnostic value in multiple cancer types. Result for prostate cancer is enclosed in the blue box (TCGA-PRAD).

Table S1. KM plotter meta-analysis results of overall survival in various cancers comparing high/low expression of AKT1 and miR-143. Only cancers for which log-rank $p < 0.05$ are presented. HR estimated for high compared to low expression (Cox proportional analysis, auto-selected cut-off).

	Cancer	***n***	*HR**	*Log-rank p-value*
AKT1	Breast cancer	1089	1.45	0.0308
	Cervical squamous cell carcinoma	304	1.69	0.0427
	Oesophageal Adenocarcinoma	80	0.26	0.0001
	Head-neck squamous cell carcinoma	499	1.42	0.0098
	Kidney renal papillary cell carcinoma	287	0.45	0.0292
	Liver hepatocellular carcinoma	370	1.7	0.0025
	Lung adenocarcinoma	504	0.7	0.0202
	Ovarian cancer	373	0.65	0.0031
	Pancreatic ductal adenocarcinoma	177	0.6	0.0305
miR-143-3p	Bladder Carcinoma	408	1.75	0.0002
	Head-neck squamous cell carcinoma	522	1.37	0.0196
	Kidney renal clear cell carcinoma	516	0.71	0.0224
	Kidney renal papillary cell carcinoma	290	2.77	0.0005
	Pancreatic ductal adenocarcinoma	178	2.06	0.0007
	Sarcoma	259	0.61	0.0334

* Red text indicates HR > 1; high expression of miR-143 or AKT1 predicted poor prognosis; blue text indicates HR < 1; low expression of miR-143-3p or AKT1 predicted favourable prognosis. *n* = number; HR = Hazard ratio; KM = Kaplan-Meier
